# ANXA1 Enhances the Proangiogenic Potential of Human Dental Pulp Stem Cells

**DOI:** 10.1155/2024/7045341

**Published:** 2024-10-23

**Authors:** Xiaocao Ma, Bichun Zhao, Chao Wang, Manqiang Sun, Yawen Dai, Lingling E., Mingzhu Gao, Xiangwei Liu, Yali Jia, Wen Yue, Hongchen Liu

**Affiliations:** ^1^School of Medicine, Nankai University, Tianjin 300071, China; ^2^Institute of Stomatology and Oral Maxilla Facial Key Laboratory, Chinese PLA General Hospital, Beijing 100853, China; ^3^Stem Cell and Regenerative Medicine Lab, Beijing Institute of Radiation Medicine, Beijing 100850, China

## Abstract

Dental trauma is highly prevalent in children and adolescents, alongside tooth decay. This condition mainly induces pulp contamination, pulp necrosis, and tooth avulsion in the clinical context. The disturbance to root growth is prone to occur in immature permanent teeth. However, conventional endodontic treatment may not achieve favorable outcomes in these cases, necessitating conducting relevant exploration. Therefore, this study was performed to examine the impact of Annexin A1 (ANXA1) on the vascular repair of dental pulp using human dental pulp stem cells (DPSCs). Specifically, RNA sequencing (RNA-Seq) and functional clustering analyses were employed to identify key genes involved in pulp regeneration. ANXA1 was detected in DPSCs and may correlate with pulp restoration. However, it remains undefined about the potential of ANXA1 to promote the angiogenetic differentiation of DPSCs. The results of this study revealed that the addition of ANXA1 significantly enhanced the secretion of vascular endothelial growth factor-A (VEGF-A) in DPSCs. Moreover, the incubation of DPSCs with ANXA1 resulted in a higher expression level of endothelial markers and promoted vessel formation through the upregulation of the phosphorylated p38 (p-p38) pathway. The in vivo results corroborated that the ANXA1 group exhibited more blood vessels and an increased ratio of positive staining for CD31. In conclusion, these findings indicate that ANXA1 enhances the in vivo and in vitro vascularization of DPSCs, and the activation of p-p38 may play a pivotal role in mediating the differentiation process.

## 1. Introduction

According to the International Association of Dental Traumatology (IADT) guidelines, traumatic dental injuries (TDIs) are highly prevalent in children and adolescents [1]. This condition often results in pulp blood and nerve losses and impairs root development in immature permanent teeth [2]. Root canal treatment is the most commonly used therapy for dental pulp injuries, and it can mechanically and chemically remove pulp tissues and eliminate symptoms [3, 4]. However, this therapy may induce the loss of pulp vitality and root development, and it is difficult to achieve self-repair due to its special physiological environment [5]. Therefore, there is an urgent demand for restoring pulp tissues and maintaining root development in traumatic immature permanent teeth in clinical practice.

Tissue engineering technology consisting of stem cells, growth factors, and scaffolds provides new opportunities for dental pulp regeneration [6, 7]. Dental pulp stem cells (DPSCs) have achieved favorable outcomes owing to their strong multi-directional differentiation ability [8]. In addition, the reasonable addition of growth factors can promote the differentiation of DPSCs and improve tissue repair efficiency [9]. Murakami et al. [10] employed granulocytic-colony stimulating factors to stimulate DPSCs and expedite vascularization in an ectopic tooth. Besides, Zhang et al. [11] demonstrated in vivo that platelet-derived growth factor-BB (PDGF-BB) effectively stimulated dentin-pulp tissue regeneration through stem cell-based approaches. Therefore, the combination of DPSCs and growth factors is a promising method for improving regeneration efficiency.

The regeneration of dental pulp vessels is a precondition for the reconstruction of the intramedullary nerve and dentin [6, 12]. In the early stage of tooth germ growth, the newly formed vascular structure in the dental papilla supplies oxygen to the whole germ tissue, which is the basis for its development [13–15]. Besides, a well-functioning blood supply ensures the nutrient supply, gas exchange, and waste removal of the tooth pulp, thus maintaining the toughness of the tooth in a physiological state [16]. When the tissue is damaged, the pulp blood vessels also play an important role in immune defense and the repair of the pulp and dentin complex [14].

As revealed in previous studies, Annexin A1 (ANXA1) may promote blood vessel formation and tissue repair. On the one hand, ANXA1 has a direct effect on inducing the polarization of myocardial macrophages to proangiogenic and repair phenotypes [17]. On the other hand, ANXA1 can also promote the release of vascular endothelial growth factor-A (VEGF-A) from human umbilical vein endothelial cells (HUVECs) to participate in the formation of blood vessels in vitro, enhance cell viability, and promote wound healing [18]. Therefore, ANXA1 is a growth factor with a strong angiogenic induction ability. However, the effect and the mechanism of ANXA1 on DPSCs in pulp angiogenesis remains poorly understood.

The p38 mitogen-activated protein kinase (MAPK) family plays an important role in regulating the proliferation, differentiation, migration, and survival of cells and the production of inflammatory mediators [19]. In a mouse model, p38 regulates the angiogenesis process through the phosphorylation of p38-regulated/activated protein kinases [20]. Furthermore, VEGF-induced endothelial migration and angiogenesis are partially dependent on the activation of the p38/mitogen-activated protein kinase-activated protein (MAPKAP) kinase-2/LIM domain kinase (LIMK1) signaling axis [21]. Recently, it has been reported that p38 may also promote angiogenesis by regulating the proteolytic activity of matrix metallopeptidase-9 (MMP-9) and MMP2 [22, 23]. In conclusion, the activation of p38 in endothelial cells (ECs) leads to actin remodeling and angiogenesis, thus playing a central role in the development process. However, it remains unclear about the role of this pathway in regulating the ANXA1-induced vasculogenic differentiation of DPSCs.

This study was conducted to evaluate whether ANXA1, in conjunction with DPSCs, could effectively promote pulp vessel repair and explore relevant mechanisms in regulating the vasculogenic fate of DPSCs.

## 2. Materials and Methods

### 2.1. Cell Culture

The experiments were approved by the Medical Ethics Committee of PLA (Approval No. S2018-094-01). This study involved the isolation of DPSCs derived from premolars or third molars of adult individuals (18–60 years old). The DPSCs were cultured and characterized as per the procedure in a previous report [24]. After four to six passages, the DPSCs were utilized to investigate angiogenetic differentiation. More specifically, the DPSCs were cultured with the MEM-alpha medium (Gibco) containing 10% fetal bovine serum (FBS; Gibco), supplemented with recombinant human ANXA1 (rhANXA1) (Cloud-Clone, China). To determine the concentration of ANXA1 to be used in subsequent experiments, the angiogenesis ability of these DPSCs treated with various concentrations of ANXA1 (10 and 20 nM) was determined by the real-time quantitative polymerase chain reaction (RT-qPCR) and enzyme-linked immunosorbent assay (ELISA). Finally, 20 nM ANXA1 was selected to induce DPSCs for 48 h given its stronger ability.

### 2.2. RNA Sequencing (RNA-Seq) Data and Gene Ontology (GO) Analysis

The sequence data utilized in this study were obtained from our previous experiments and had been deposited in the Gene Expression Omnibus (GSE185751, https://www.ncbi.nlm.nih.gov/geo/). RNA-seq was conducted on the DPSCs, and 18,801 messenger RNAs (mRNAs) were obtained [24]. The GO functional clustering analysis of these mRNAs was conducted using the bioinformatics platform of the Database for Annotation, Visualization and Integrated Discovery (DAVID) of the National Institutes of Health (NIH) (https://david.ncifcrf.gov/). GO terms with a *p*-value less than 0.05 were deemed significantly enriched and sorted in ascending order. The Venn diagram was plotted to display the GO terms related to dental pulp repair. Moreover, the genes related to proliferation, negative regulation of apoptosis, and inflammation were further analyzed to determine the key genes.

### 2.3. RT-qPCR

Total RNA extraction was performed using Trizol (Ambion) following the manufacturer's instructions. Subsequently, complementary DNA (cDNA) synthesis was carried out using an RT Master Mix (Toyobo). The expression levels of relevant genes, such as VEGF-A and MMP-3, were quantified, with *β*-actin serving as the housekeeping gene for RNA expression normalization. The PCR primer sequence is listed in Table 1.

### 2.4. ELISA for the In Vitro Production of VEGF-A

After incubation with ANXA1 for 48 h, the supernatant was collected from each sample. The concentration of VEGF-A was quantified using a VEGF-A ELISA kit (Elabscience) in accordance with the manufacturer's instructions. The data were standardized based on the total protein content.

### 2.5. Cell Proliferation Analysis

To assess the impact of 20 nM ANXA1 on cellular proliferation, the cell counting kit-8 (CCK-8, Dojindo) assay was conducted. Cell suspensions containing 2000 cells per well were added to 96-well plates at a volume of 100 μL per well. After cell attachment, these DPSCs were subjected to treatment with 20 nM ANXA1 in minimum essential medium *α* (*α*-MEM) supplemented with 10% FBS. At specified time intervals, 10 μL of CCK-8 solution was introduced into each well, and the cells were incubated at 37°C in a 5% CO_2_ environment for 2 h. The absorbance was subsequently measured at 450 nm using a SpectraMax M2 instrument (Molecular Devices).

### 2.6. Evaluation of DPSCs Migration by the Transwell Assay

In the DPSCs migration assay, Transwell inserts with 8-μm pores (Corning) were employed. Specifically, the cell suspension containing 1.2 mL of 3 × 10^5^ cells/mL was loaded into the upper chamber and treated with 1% FBS and 20 nM ANXA1 in *α*-MEM. Subsequently, 1.5 mL of *α*-MEM supplemented with 10% FBS was introduced into the lower chamber. After the incubation at 37°C for 48 h, the DPSCs located in the upper chamber were fixed using a 4% paraformaldehyde solution and subsequently stained with 1% crystal violet. The cells situated on the upper surface of the membrane were eliminated, and the video image of DPSCs migration was captured. The number of migrated DPSCs within five randomly chosen microscopic fields was quantified and analyzed.

### 2.7. Flow Cytometry Analysis

To ascertain all alterations in the expression of surface marker proteins of the DPSCs, a flow cytometry analysis was conducted. The DPSCs were harvested and suspended in phosphate-buffered saline (PBS) to generate a cell suspension with a density of 1 × 10^6^ cells/mL. Subsequently, the DPSCs were incubated with EC markers, namely CD31-APC (1:100 dilution, Thermo Fisher Scientific) at 4°C for 30 min. After the staining process, the DPSCs were washed and resuspended in 300 μL of PBS. Flow cytometry was performed with the aid of the Guava easyCyte system from Luminex and ImageJ to determine the mean fluorescence intensity of the surface markers.

### 2.8. Cell Apoptosis Analysis

The influence of ANXA1 on DPSCs apoptosis was assessed using the Annexin V/PI staining assay (Annexin V, 633, Dojindo). Specifically, the DPSCs were centrifuged and resuspended in 1× binding solution at a density of 1 × 10^6^ cells/mL. Then, the DPSCs from each experimental group were incubated with Annexin V (1:100) and PI (1:100) in separate tubes at room temperature for 15 min. After being washed three times, 300 μL of binding solution was added to each tube. Finally, the DPSCs were analyzed using a flow cytometry analyzer.

### 2.9. Matrigel-Based Vessel-Like Tube Formation Assay In Vitro

To initiate the experiment, precooled 96-well plates were coated with growth factor reduced (GFR) Matrigel (Corning) and incubated at 37°C for 1 h. Then, the DPSCs were cultured with or without ANXA1 for 48 h, followed by the suspension in EGM2-MV (Lonza) before being seeded into 96-well plates at a density of 3 × 10^4^ cells per well. Subsequently, the plates were incubated at 37°C in a 5% CO_2_ atmosphere for 7 h. The formation of vascular-like structures was observed using a light microscope (ECLIPSE-TS2, Nikon), and the images were analyzed by the Angiogenesis Analyzer plugin of ImageJ. The total number of junctions and total length of vessels were determined as parameters indicative of tube formation efficacy.

### 2.10. Western Blot (WB) Analysis

To measure the involvement of the p38 MAPK pathway, the adenosine triphosphate (ATP)-competitive inhibitor Adezmapimod (SB203580, 20 μm) was used. Besides, the WB analysis was performed under three different conditions for 48 h:

1. DPSCs were treated with 10% FBS;
2. DPSCs were incubated with 20 nM ANXA1 + 10% FBS;
3. DPSCs were pretreated with SB203580 + 20 nM ANXA1 + 10% FBS.

After treatment, the total protein from different groups was harvested using the radio immunoprecipitation assay lysis buffer (RIPA) Lysis Buffer (CWBIO) supplemented with protease inhibitors (1:100; Bimake) and phosphatase inhibitors (1 : 100; Bimake) and then placed on ice for 50 min. Subsequently, the cell lysate was subjected to centrifugation at a speed of 12,000 revolutions per minute under 4°C for 10 min. The total protein concentration was determined using a bicinchoninic acid (BCA) protein assay kit. Next, the cell lysates in equal amounts were applied to the precast gel (Tsingke) and transferred to the polyvinylidene difluoride (PVDF, Millipore) membrane with a pore size of 0.22 µm. The membranes were subsequently blocked with a 5% nonfat milk solution and incubated overnight at 4°C with primary antibodies against p38, phosphorylated p38 (p-p38), and *β*-actin (R&D Systems). After that, the membranes were incubated with secondary antibodies (Biosharp) conjugated with horseradish peroxidase (HRP). Finally, the protein bands were observed using the Gel imaging system (GE HealthCare).

### 2.11. Histological Immunohistochemical Analyses In Vivo Implantation

The animal study protocol was approved by the Academy of Military Medical Sciences Laboratory Animal Center. To assess dental pulp vascular regeneration, a subcutaneous ectopic transplantation model of the human tooth slice was established. Human molars were cut into slices of 1.5 mm in length. Then, the enamel and pulp tissues were removed. Subsequently, the tooth slices were concussed in deionized water for 30 min by an ultrasonic cleaner and then exposed to 70% ethanol solution for disinfection. Next, 1 × 10^6^ DPSCs (passages 4–6) without any treatment, 70 μL Matrigel, and 1 μM human recombinant ANXA1 protein were injected into the pulp cavity of tooth slices, and whole-body transplantation was performed subcutaneously in mice for culture.

Based on these tooth slices, this experiment was divided into three groups as follows:

1. Control: Matrigel;
2. DPSCs: DPSCs + Matrigel;
3. DPSCs + ANXA1: ANXA1 + DPSCs + Matrigel;

The severe combined immunodeficiency (SCID) mice (6 weeks old) were used for transplantation. The procedure was performed under general anesthesia with isoflurane. The fur on the back of mice was shaved, followed by cleaning and sterilization with povidone-iodine. Then, a midsagittal incision was made dorsally, and one or two subcutaneous pockets were made using blunt dissection. Subsequently, five mice in each group were implanted with eight tooth slices, and a total of 15 mice were used. Next, the samples were implanted in each group, and the incision was closed with staples after the tooth slices were placed properly. About 5 weeks after transplantation, these mice were euthanized as per the protocol approved by the Institutional Animal Care and Use Committee (IACUC). The back of the mice was cut open with a sharp instrument, and the samples were gently removed. The harvested samples were immediately fixed in 10% formalin for 24 h and subjected to decalcification in a 10% ethylene diamine tetraacetic acid (EDTA) solution for 1 week. Finally, the posttreatment samples were embedded in paraffin wax and sectioned in a thickness of 5 µm, followed by hematoxylin and eosin (HE) staining. Additionally, the percentage of blood vessel tissue area within the entire implant was calculated. To analyze matrix formation, every eight paraffin sections from different groups were stained with Masson's trichrome (Solarbio).

### 2.12. Immunohistochemical and Immunofluorescence Analyses In Vivo Implantation

To verify whether the regenerated vascularization and implanted DPSCs completed the self-differentiation into the reconstituted pulp, immunohistochemical staining was performed on the sections with CD31, EC biomarker, and human antinucleolus antibody (ANCAb). Specifically, the samples from each group were deparaffinized and subjected to antigen retrieval in 0.01 M citrate buffer (PH = 6) at 95°C for 35 min. Then, immunohistochemistry was performed using the Universal two-step assay kit + diaminobenzidine (DAB) color kit (ZSGB-Bio). Subsequently, the endogenous peroxidase was blocked with the peroxidase block included in the kit for 15 min. Next, the sections were washed three times in PBS for 5 min and permeabilized with 0.2% Triton X-100 for 30 min. After the permeabilization, the sections were washed for 5 min in PBS and blocked with 10% goat serum (Beyotime) for 60 min. The sections were incubated in a primary antibody (CD31, 1:100 dilution, R&D Systems; ANCAb, 1:200, Merck) overnight at 4°C. After the incubation, the sections were washed in PBS and incubated for 1 h at room temperature with the secondary antibodies (1:400, Abcam). Then, the sections were washed three times in PBS for 5 min and treated with the DAB chromogenic agent for 5 min. Subsequently, the material was washed in PBS for 5 min and stained with Mayer-hematoxylin for 30 s. After that, the sections were rinsed with water, followed by dehydration, transparency, and sealing using neutral resin. The images were captured and the area fraction of CD31 positive capillaries to the root canal area was determined using ImageJ.

The regenerated tissue was fixed, sectioned, sealed, and ruptured as described above. Then reconstituted pulps raised in SCIDs were incubated with primary antibody CD31 (goat antihuman/mouse/rat, 1:100 dilution, R&D Systems) and human nuclei (mouse antihuman, ANCAb, 1 :200, Merck) overnight at 4°C. Slices were washed in PBS and stained with secondary antibodies for 1 h at room temperature, avoiding light. Secondary antibodies used included donkey anti-goat-fluorescein isothiocyanate (FITC) (green, 1:400, Abcam) and donkey anti-mouse-Texas-Red (red, 1:400, Abcam). The sections were washed again with PBS for 5 min and sealed using 90% glycerin. Images were acquired using the Olympus Fluoview FV1000 laser scanning microscope (Olympus) and determined by ImageJ.

### 2.13. Statistical Analysis

All experiments were performed three times independently. The experimental data were expressed as mean ± standard deviation (SD). The differences among data sets were analyzed using the analysis of variance (ANOVA) or Student *t*-test. Statistical significance was determined by the *p*-value less than 0.05.

## 3. Results

### 3.1. ANXA1 Was Identified to be Involved in Pulp Repair

**Figure 1:**
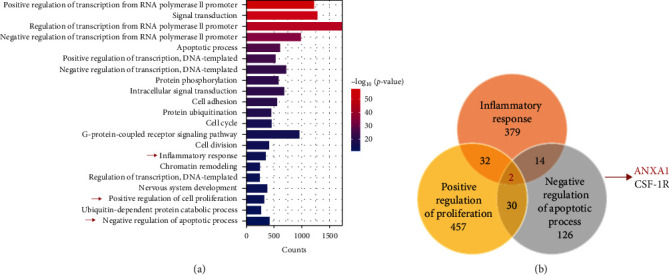
Identification of ANXA1 candidate target genes related to dental pulp regeneration. (a) GO biological process enrichment analysis of DPSCs (*p*  < 0.05). (b) Venn diagram shows the intersection genes of GO terms. ANXA1, Annexin A1; CSF-1R, colony-stimulating factor 1 receptor; DPSCs, dental pulp stem cells; GO, gene ontology.

To obtain the key genes related to dental pulp repair, the whole transcriptome gene sequencing analysis was performed on human DPSCs in a physiological state. Eventually, 18,801 mRNAs were obtained. These mRNAs were subsequently subjected to the GO enrichment analysis to confirm the function of these genes. In the biological process classification, the top-ranked items related to dental pulp repair were concentrated (Figure 1a). The genes in these terms were overlapped, and the Venn diagram was plotted to obtain ANXA1 and colony-stimulating factor 1-receptor (CSF-1R). Since the average read count of ANXA1 was 9706 and the average read count of CSF-1R was 39 in sequencing, ANXA1 was identified for subsequent experiments (Figure 1b).

### 3.2. The Angiogenesis Ability of DPSCs Was Promoted by ANXA1.

**Figure 2:**
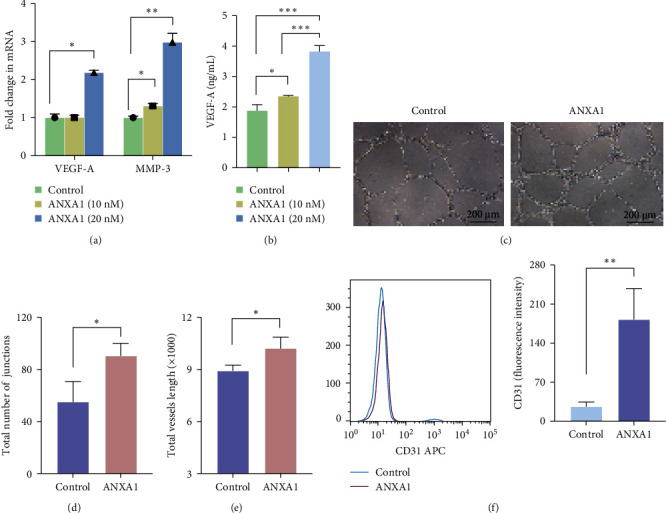
ANXA1 promotes the angiogenic ability of DPSCs. (a) mRNA expression levels of VEGF-A and MMP-3 after 48 h induction. mRNA levels were normalized to the housekeeping control gene *β*-actin. (b) The levels of VEGF-A secreted by DPSCs at different ANXA1-induction concentrations. (c) Representative images of induced DPSCs in Matrigel. (d) The junction's number and (e) the total tube length of meshes. (f) Mean fluorescence intensity was recorded in flow cytometry assay. For all graphs *n* = 3 and quantification from three independent experiments (*⁣*^*∗*^*p*  < 0.05; *⁣*^*∗∗*^*p*  < 0.01; *⁣*^*∗∗∗*^*p*  < 0.001). ANXA1, Annexin A1; DPSCs, dental pulp stem cells; MMP-3, matrix metalloproteinases 3; VEGF-A, vascular endothelial growth factor A.

The rhANXA1 with different concentrations (10 and 20 nM) was added to the culture medium to examine the effect of rhANXA1 on DPSCs. The expression levels of three genes, namely VEGF-A, MMP-3, and the secretion of VEGF-A protein, were analyzed. After the stimulation with 20 nM rhANXA1, the gene expression levels of VEGF-A and MMP-3 increased by 2.2 and 3.0 folds, respectively (Figure 2a). Furthermore, the protein secretion level of VEGF-A in the cell supernatant significantly increased by a factor of 2.0 after the stimulation with high-concentration rhANXA1 (Figure 2b). Hence, 20 nM was determined to be the optimal concentration of rhANXA1 for all subsequent experiments.

Given the potential of VEGF-A secreted by DPSCs to enhance vascularization, a more specific angiogenesis assay was conducted. Induced and uninduced DPSCs were seeded into 96-well culture plates coated with Matrigel and cultured with EGM2-MV for 7 h. The induced group exhibited a larger number of junctions and total length compared with the control group (Figure 2c–e). The expression of cell surface markers was further examined after the incubation with rhANXA1. Figure 2f illustrates a significant enhancement of the mean fluorescence intensity in the EC marker CD31 in the rhANXA1 group compared with the control group.

### 3.3. The Angiogenic Activity of rhANXA1 on DPSCs *via* p-p38 Signaling

**Figure 3:**
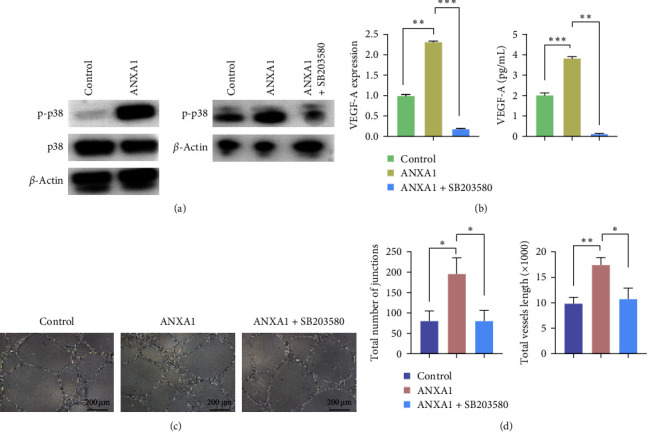
ANXA1 stimulates angiogenic ability of DPSCs through the p-p38 signaling pathway. (a) Representative WB images illustrated the differential expression of p-p38/p38 after treatments and p38 inhibitors' effect on DPSCs. (b) SB203580 attenuated ANXA1-induced VEGF-A mRNA expression and protein production. (c) Angiogenesis of DPSCs to p-p38 inhibitor, Matrigel assay, number of junctions, and total vessel length (d). For all graphs *n* = 3 and quantification from three independent experiments (*⁣*^*∗*^*p*  < 0.05; *⁣*^*∗∗*^*p*  < 0.01; *⁣*^*∗∗∗*^*p*  < 0.001). ANXA1, Annexin A1; DPSCs, dental pulp stem cells DPSCs; p-p38, phospho-p38; VEGF-A, vascular endothelial growth factor A; WB, western blot.

After 48 h of culture, the protein expression level of the phospho-p38 MAPK pathway was observed to determine the pathways involved in the angiogenesis of DPSCs.  Figure 3a shows upregulated p-p38 upon induction (no significant difference in p38), indicating the activation of the p-p38 pathway in rhANXA1-induced angiogenesis. To conduct further investigation, the DPSCs were pretreated with Adezmapimod (SB203580, 20 μM), a selective and ATP-competitive p38 MAPK inhibitor, to inhibit the signaling pathway. In this study, the downregulation of mRNA and the supernatant protein expression level of VEGF-A were also observed, as depicted in Figure 3b. These findings were corroborated by the statistical analysis of tube formation, aligning with the data obtained from the RT-qPCR and ELISA. Specifically, the incubation with the p-p38 inhibitor resulted in a significant decrease in the number of junctions and tube length in stimulated samples, as illustrated in Figure 3c,d.

### 3.4. Maintenance of the Proliferation, Apoptosis, and Migration Ability of DPSCs

**Figure 4:**
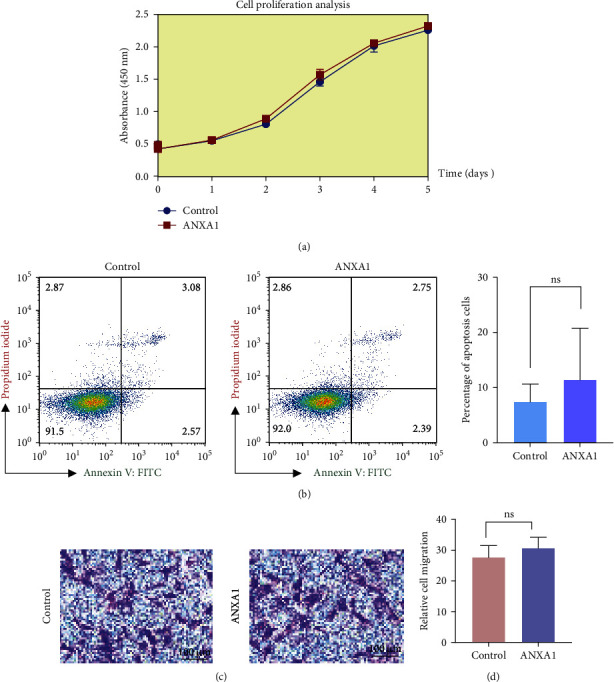
Effect of ANXA1 on proliferation, apoptosis, and migration of DPSCs. (a) DPSCs proliferation was detected by CCK-8. (b) Annexin V-FITC/PI staining to evaluate apoptosis. (c, d) DPSCs transwell migration assay. For all graphs *n* = 3 and quantification from three independent experiments. ANXA1, Annexin A1; CCK-8, cell counting kit-8; DPSCs, dental pulp stem cells DPSCs; ns, no significance.

To investigate the potential negative effects of 20 nM rhANXA1 on the proliferation, apoptosis, and migration of DPSCs, the CCK-8 kit, Annexin V/PI staining kit, and Transwell migration assay were employed in this study. It was found that the results of the three tests were comparable, and there was no significant statistical difference between the induced DPSCs and the control group. As depicted in Figure 4a, there were no discernible variations in the relative absorbance readings at 450 nm between the unstimulated and stimulated groups from Day 1 to Day 5. Similarly, the impact of 20 nM rhANXA1 on DPSCs apoptosis aligned with the above results (Figure 4b). Moreover, the migration ability was also assessed, and the results revealed no disparity in the quantity of migrated cells (Figure 4c,d).

### 3.5. Ectopic Pulp Regeneration in the Tooth Root After DPSCs Transplantation

**Figure 5:**
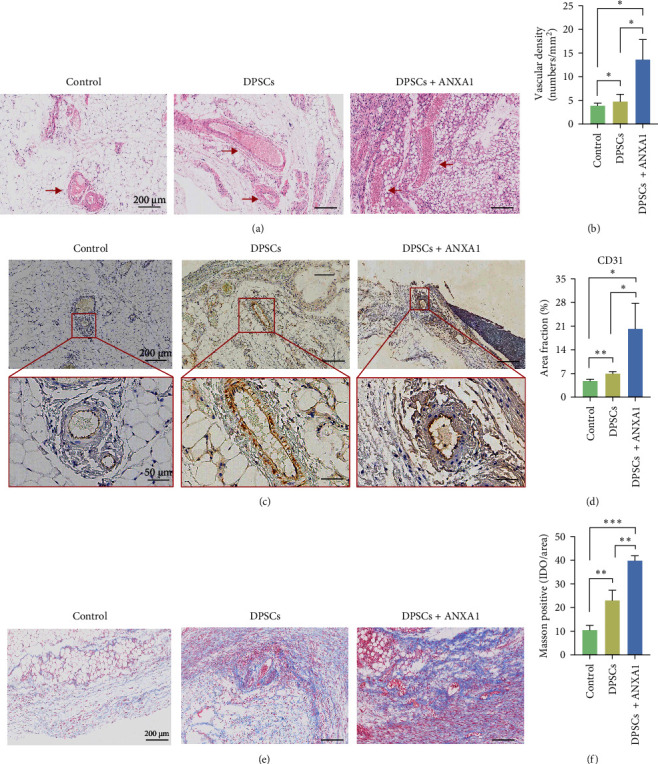
Ectopic transplantation of DPSCs contributes to pulp tissue regeneration in SCID mice. (a) HE staining. (b) Ratio of the newly regenerated area to root canal area. (c, d) Anti-CD31 immunohistochemistry and area fraction data. (e) Representative images of Masson's trichrome stain (Masson). (f) Analysis of the amount of regenerated fiber tissues. For all graphs *n* = 8 and quantification from three independent experiments (*⁣*^*∗*^*p*  < 0.05; *⁣*^*∗∗*^*p*  < 0.01; *⁣*^*∗∗∗*^*p*  < 0.001). ANXA1, Annexin A1; DPSCs, dental pulp stem cells DPSCs; HE, hematoxylin- and eosin; SCID mice, severe combined immune-deficiency mice.

The potential for pulp regeneration induced by rhANXA1 in DPSCs was measured using an ectopic tooth slice transplantation model in SCID mice. DPSCs, with or without rhANXA1, were used to regenerate pulp-like tissues in tooth slices for 5 weeks. The HE staining results confirmed the presence of blood vessels in all experimental groups (Figure 5a,b). These vessel-like tissues were surrounded by endothelium-like or smooth muscle-like cells and contained round red blood cells in the lumen. However, there was a variation in the quantity of regenerated blood vessels across different samples. The statistical analysis results suggested that the area of regenerated vascularization in the group induced with rhANXA1 was 2.8 and 3.4 times larger than that of the uninduced DPSCs and the control group, respectively.

These findings were further confirmed by the immunohistochemical staining results of CD31, an EC biomarker. The portion of the regenerated tissue stained positively for CD31 proved that the vessel wall was predominated by ECs, and the newly formed vessels were blood vessels (Figure 5c,d). Moreover, it was evident that the induced group exhibited a larger ratio of positive regions compared with the noninduced group. The Masson trichrome staining results demonstrated matrix formation in all samples, with a higher abundance of collagen in the rhANXA1-induced group (Figure 5e,f).

**Figure 6:**
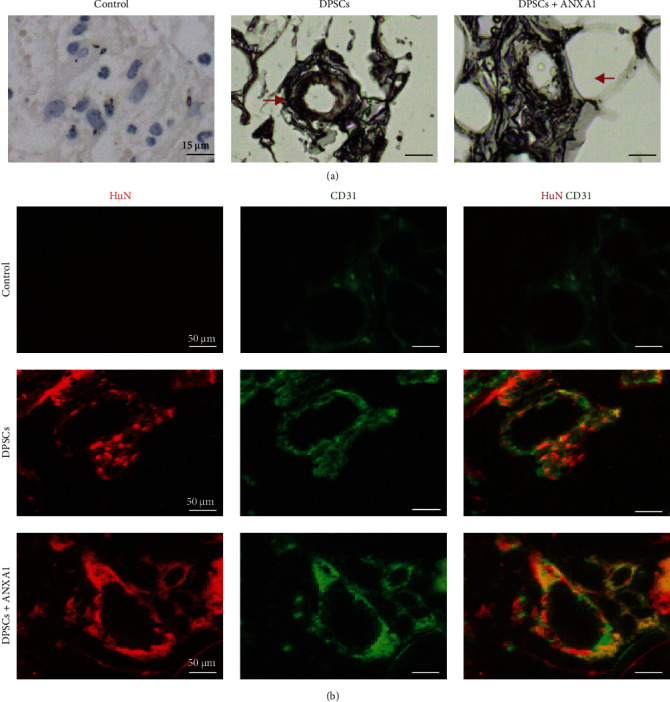
The newly formed tissues are partly derived from DPSCs in SCIDs. (a) Immunohistochemical analysis using anti-HuN antibody. Anti-HuN antibody-positive cells were observed near the newly generated vessels (red arrow). (b) Immunofluorescence staining of HuN (red), EC marker (CD31, green). ANXA1, Annexin A1; DPSCs, dental pulp stem cells; EC, endothelial cell; HuN, human nucleoli; SCIDs, severe combined immune-deficiencies.

To confirm the origin of reconstituted pulp vessels, immunohistochemical staining with human ANCAb was performed on the sections. No human cells were detected in the control group by immunostaining. However, the cells with positive antihuman nucleolar antigens were found around the newly formed vessels in the DPSCs implantation group (Figure 6a). To visualize the relationship between ECs and DPSCs more intuitively, we used double staining of human nucleoli (HuN) and CD31. Similarly to the results above, obvious HuN associated with CD31-labeled ECs can be seen in most vascular structures, and their positions are consistent in DPSCs and ANXA1 groups (Figure 6b). However, in the control group, only a small amount of CD31 antibody was positively expressed, while ANCAb was not seen.

## 4. Discussion

According to the IADT guidelines, TDIs account for 5% of all injuries. Additionally, dental trauma has been reported in 25% of school-age children and 33% of adults [2, 25]. Such sequelae as pulp discoloration and tooth loss may have long-term effects on the physical and psychological health of patients. In terms of the treatment of TDIs, particularly in immature permanent teeth, the primary objective is to maintain pulp vitality or, at the very least, preserve pulp blood vessels [26].

Dental pulp regeneration engineering encompasses three essential components, namely cells, growth factors, and scaffolds [8, 27, 28]. Among them, growth factors play a pivotal role in regulating crucial cellular processes involved in maintaining pulp equilibrium and tooth development through the activation of various signaling pathways. Notably, previous studies have demonstrated the ability of basic fibroblast growth factors to stimulate the *in vitro* proliferation, differentiation, and matrix synthesis of DPSCs [29]. Nam et al. [30] observed that stromal cell-derived factor-1*α* (SDF-1*α*) facilitated the angiogenesis of DPSCs through the SDF-1*α*/CXCR4 axis in some *in vivo* experiments. Overall, a strong growth factor produced by DPSCs, which is integral to the repair process, is essential for relevant applications in clinical practice.

In this study, the RNA-seq assay was performed on adult DPSCs, and ANXA1 was identified as a gene closely associated with dental pulp repair functions. Besides, relevant experiments on DPSCs were conducted to evaluate the angiogenetic regeneration ability of ANXA1 by measuring vessel formation and related indicators. ANXA1 has been widely recognized for its significant contributions to the innate immune system as an effector of glucocorticoid-mediated responses and a regulator of the inflammatory process [31, 32]. Additionally, ANXA1 is involved in blood vessel formation and actin migration, thereby mitigating inflammation and promoting wound healing [33, 34]. Pessolano et al. [18] demonstrated that mesoglycan induced proangiogenic effects by facilitating the interaction between extracellular vesicles carrying ANXA1 and formyl peptide receptors, thus resulting in the release of VEGF-A and the subsequent promotion of vascularization. Ferraro et al. [17] confirmed that ANXA1 exerted a direct impact on cardiac macrophages, leading to the production of VEGF-A in large amounts and their involvement in neovascularization and cardiac repair. In this study, the increased secretion of VEGF-A by DPSCs was observed upon ANXA1 stimulation, which was consistent with the above findings. Consequently, VEGF-A was validated to play a crucial role in the angiogenesis induced by ANXA1.

DPSCs constitute an essential part in the regeneration of dental pulp blood vessels. There are two hypotheses indicating that DPSCs are involved in sprouting angiogenesis. On the one hand, some studies have suggested that DPSCs can even differentiate into endothelial-like cells and contribute to the formation of new blood vessels [35–37]. It should be noted that the endothelial phenotypic identification and vessel structure formation in Matrigel assays provide solid proof for the differentiation of DPSCs into ECs. In this study, the results directly proved the higher expression of CD31 and the stronger ability to form capillaries *in vitro*. More importantly, the positive expression of human nucleoprotein in the wall of regenerated blood vessels in animal experiments provided more intuitive evidence that the endothelial tissue in the dental pulp cavity originated from the differentiation of human DPSCs. Therefore, these DPSCs treated by ANXA1 exhibited a higher endothelial differentiation potential. On the other hand, the pericycle-like effects of DPSCs have been demonstrated in many studies. More specifically, they can guide ECs in a paracrine manner and support angiogenesis by adopting pericytial positions to stabilize newly formed blood vessels [38–40]. However, this hypothesis should be considered with caution in this study. For one thing, pericytoma identification usually relies on cytoplasmic and membrane markers, such as alpha-smooth muscle actin (*α*SMA), nestin, desmin, tropomyosin, PDGF receptor-*β*, and nerve/glial antigen-2 proteoglycan [41]. For another, the VEGF is known to be a negative inhibitor of pericytes; it will ablate pericyte coverage of vascular sprouts, leading to vessel destabilization and disrupting pericyte recruitment during blood vessel formation [42, 43]. Therefore, it may be inferred that upregulated VEGFs in DPSCs interfere with the differentiation of pericytes in this research. However, further research is required to fully establish their role in pericyte-like functions. It is worth noting that the regeneration of blood vessels and collagen was also observed in samples without DPSCs implantation, which is derived from cell migration and differentiation in mice, but the specific ability and strong evidence need to be further studied.

**Figure 7:**
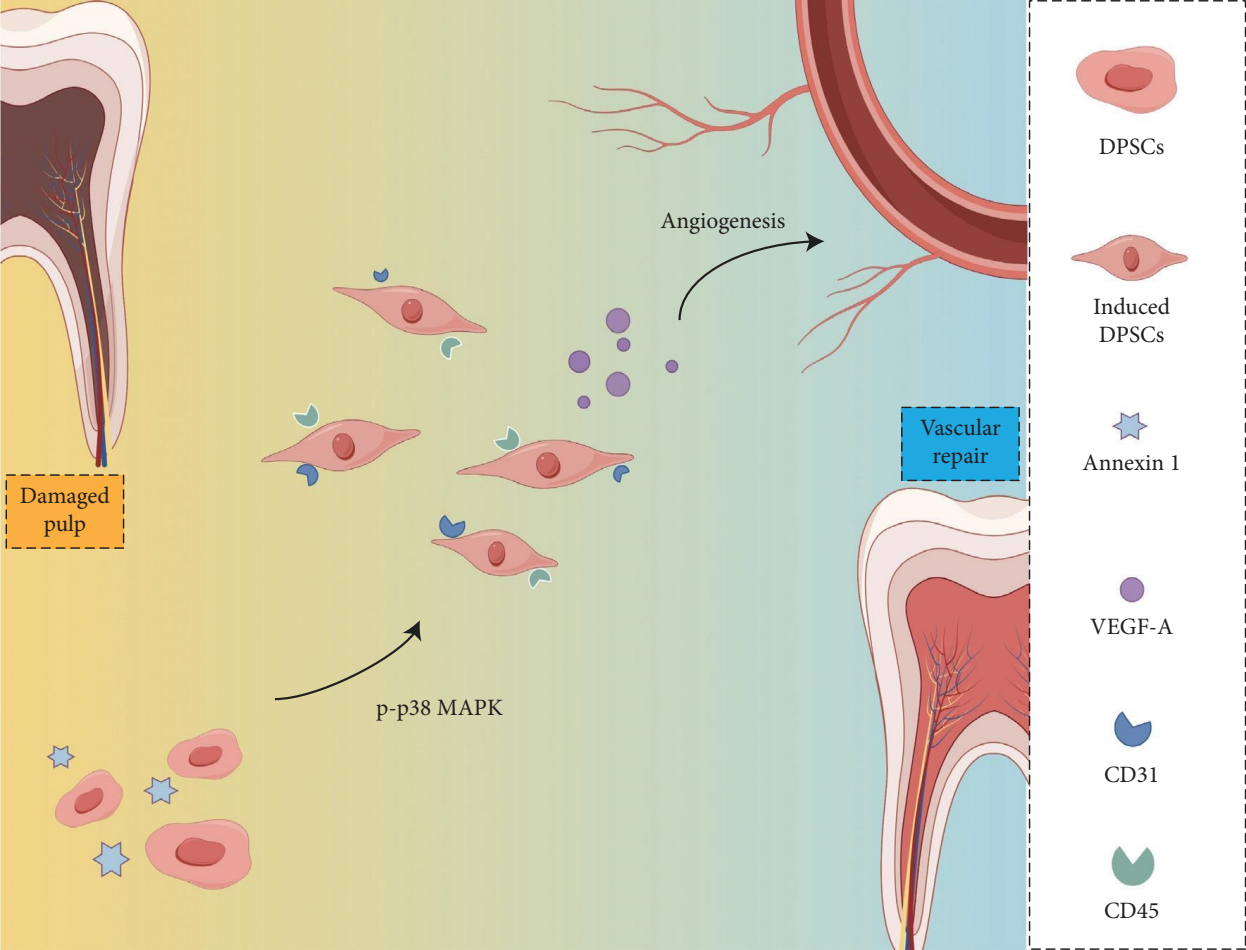
A possible network of molecular events targeted by ANXA1 related to angiogenesis differentiation of DPSCs in vitro. Stimulation of DPSCs by ANXA1 may result in overexpression of p-p38 MAPK and then the activation of VEGF-A. Besides, the interaction and crosstalk among ANXA1, p-p38 MAPK, and VEGF-A will regulate the angiogenesis differentiation of DPSCs. ANXA1, Annexin A1; DPSCs, dental pulp stem cells; MAPK, mitogen-activated protein kinase; p-p38, phospho-p38; VEGF-A, vascular endothelial growth factor A.

In the context of dental pulp growth and development, the epithelium can regulate mesenchymal cells through signal transmission [44, 45]. The p38 MAPK pathway plays a crucial role in regulating various cellular processes, including proliferation, migration, and odontogenic and angiogenesis differentiation of DPSCs. Inhibiting p38 MAPK decreases the secretion of angiogenic-dependent proteins and prevents the angiogenic behavior of DPSCs cultured on minerals [46, 47]. The P38 signaling pathway mediates the production of VEGFs under the induction of hypoxia-inducible factor a, thus increasing the angiogenic potential of DPSCs [48]. In ECs, p38 activated by DPSCs facilitates sprouting angiogenesis [49]. Furthermore, the activation of the p38 pathway has been shown to modulate the expression of VEGFs and angiogenesis under the induction of tumor necrosis factor-alpha and leptin [50, 51]. Hence, the significant contribution of p-p38 in the angiogenic process of DPSCs can be demonstrated based on these findings. The WB and angiogenesis assay results corroborated that the effect of ANXA1 was nullified upon the addition of the p38 inhibitor. These results proved that ANXA1 can enhance the angiogenesis of DPSCs by activating p-p38 signaling and upregulating VEGF-A (Figure 7).

Although it has been confirmed that the combination of ANXA1 and DPSCs may be an effective approach to pulp blood regeneration, it remains unclear whether ANXA1 is useful for the pulp nerve and dental pulp–dentine complex regeneration. Doubtedly, the role of ANXA1 in promoting cell proliferation and migration in GO prediction was not realized in vitro experiments, which may be related to the prerequisites in the angiogenic process. The concentration of ANXA1 plays a major role in the regulation of cell fates, which may explain various functions observed under the same growth factor. In this study, it was validated that the implantation of ANXA1 and DPSCs was an effective method for promoting pulp regeneration. However, the newly formed tissues are disordered and different from the well-organized structure in the dental pulp. The combination with other growth factors or scaffolds should be explored in future studies.

## 5. Conclusions

The results of this study demonstrate that the administration of ANXA1 enhances the angiogenic differentiation ability of DPSCs, which can be proved by the increased secretion of VEGF-A and the expression of EC markers. Furthermore, the activation of the p-p38 MAPK pathway is identified as a critical mediator in the angiogenic effect. These findings may establish an advanced approach to dental pulp engineering and provide a basis for the subsequent restoration of the pulp-nerve and dentin complex.

## Figures and Tables

**Table 1:** Nucleotide sequences for real-time RT-PCR primers.

Gene	Primer sequence (5′−3′; forward/reverse)	Product size (bp)	Accession number
VEGF-A	AGGGCAGAATCATCACGAAGT	75	NM_001171627
	AGGGTCTCGATTGGATGGCA		

MMP3	AGTCTTCCAATCCTACTGTTGCT	226	NM_002422
	TCCCCGTCACCTCCAATCC		

*β*-Actin	CATGTACGTTGCTATCCAGGC	250	NM_001101
	CTCCTTAATGTCACGCACGAT		

Abbreviations: MMP3, matrix metalloproteinases 3; VEGF-A, vascular endothelial growth factor A.

## Data Availability

RNA sequencing data are available on Gene Expression Omnibus GSE185751.
